# Updates on the polio surveillance action plan's key performance indicators: East and Southern African countries (January 2022 to December 2023)

**DOI:** 10.11604/pamj.2025.50.85.43108

**Published:** 2025-03-26

**Authors:** Daudi Manyanga, Charles Byabamazima, Brine Masvikeni, Sarah Wanyoike

**Affiliations:** 1 World Health Organization, Inter-Country Support Team, Office for East and Southern Africa, Belvedere, Harare, Zimbabwe

**Keywords:** Polio eradication, surveillance, poliovirus, acute flaccid paralysis

## Abstract

The Global Polio Eradication Initiative (GPEI) partners are closely monitoring the progress of the implementation of key strategic activities of the polio eradication strategy for the years 2022-2026. This is being done using the Global Polio Surveillance Action Plan 2022-2024, which includes a set of indicators to track progress. All countries in the ESA sub-region have been informed about these key performance indicators, and the World Health Organization (WHO) is keeping a weekly eye on their implementation. We conducted a descriptive secondary analysis of field Acute Flaccid Paralysis (AFP) surveillance data compiled from 19 countries of East and Southern Africa for the period of January 2022 to December 2023. During the period, Seychelles did not report any AFP case, and therefore the country is excluded from the final analysis. A total of 18,078 AFP cases were detected, reported, and verified from nineteen countries in ESA and we found 8,049 females (44.5%) and 10,029 males (55.5%). This shows a male child was more likely to get AFP compared to a female child (11% difference, p < 0.0001). We noticed a significant increase in prompt AFP cases detection of 4% and an increased number of cases investigated within 11 days from the date of onset of paralysis from 7,790 (89%) in 2022 to 8,246 (91%) in 2023 (2% change, p < 0.0001). The two AFP surveillance quality indicators in the study period rose from 4.4 in 2022 to 4.7 in 2023 (0.3 increase, p < 0.0001). Of the 8,964 AFP stool samples assessed in 2023, 53% were delivered to the national polio laboratories within 72 hours compared to 52% of 8,666 AFP stool samples in 2022 (1% difference, p-value of 0.1838). There is an overall improvement in the Global Polio Surveillance Action Plan (GPSAP) indicators in the ESA subregion for the period of 1 January 2022 to 31 December 2023. We recommend utilizing innovative methods to improve case detections, providing tailored surveillance training to lower levels, and enhancing sample shipment and capacities to process samples timely.

## Introduction

Poliomyelitis is one of the vaccine-preventable diseases affecting mostly children under-five years of age and the disease was selected for global eradication [1,2]. Since the World Health Assembly (WHA) meeting in 1948 that resolved global polio eradication, several steps and strategies have been implemented so far [3]. The initial strategy was the formation of the Global Polio Eradication Initiative (GPEI) in 1988 and the development of the polio eradication strategy from 1988 to 2000 with the goal of eradicating the disease by 2000 [4]. By the end of the first strategy in 2000, a dramatic 99% decline in wild polio cases was observed from over 350,000 cases in 1988 to less than 3500 (2000) in addition to the reduction of polio-endemic countries from 125 (1988) to only 20 by the year 2000 [5]. Among the challenges that were reported to delay the achievement of the planned target were suboptimal vaccination campaign quality, inadequate polio surveillance structures, and routine vaccination coverage. After that, six specific period strategic plans to eradicate polio were developed for 2001-2005, 2004-2008, 2010-2012, 2013-2018, 2019-2023 and the current one of 2022 to 2026 being implemented to address the remaining challenges of polio eradication. Among the major achievements of the global polio eradication initiative was the certification of the WHO African Region on 20 August 2022, as free of the indigenous wild poliovirus in spite of the COVID-19 pandemic disruptions [6]. Nevertheless, the struggle to eradicate polio globally with refined strategies, structures, and systems discussed widely continues to have hurdles towards achieving the global polio eradication goals. Some of the commonly documented challenges include suboptimal immunogenicity of Oral Polio Vaccine (OPV) in certain high-risk settings that are inaccessible, biological risk and genetic instability, lack of political support in certain countries, social conflicts and anti-OPV rumors, challenges in introducing the Inactivated Polio Vaccine (IPV), polio transition challenges, and vaccine supply related challenges [7-10].

At the beginning of 2022, globally only two countries remained endemic to wild poliovirus (Afghanistan and Pakistan) but there was an importation of WPV1 into Malawi and Mozambique [11]. Also, an increased spread of circulating vaccine-derived poliovirus (cVDPV) was reported in three of the six WHO regions and this resulted in the alignment of the polio eradication strategy 2022-2026 into two separate goals, one for the endemic countries and the other to stop cVDPV transmission. This alignment streamlined the focus and targets in better-refined ways compared to the previous strategic plans [12].

The GPEI partners track the implementation of the polio eradication strategy by developing the Global Polio Surveillance Action Plan (GPSAP) 2022-2024 with a set of indicators to monitor progress [13]. The GPSAP comprises six overarching objectives and a set of indicators to monitor timeliness in rapidly detecting any WPV or cVDPV from Acute Flaccid Paralysis cases or environmental samples, rapidly processing the samples to generate prompt results for rapid outbreak response. In addition, environmental surveillance is also implemented in most of the WHO African countries to supplement AFP surveillance [14]. All countries in the ESA subregion were oriented on the GPSAP 2022-2024 key performance indicators in 2022 and the WHO in the subregion is monitoring their implementation every week.

This publication aimed to document the progress of the field polio surveillance using GPSAP indicators in the ESA countries for the period of January 2022 to December 2023. In addition, it provides an opportunity to show and discuss key areas that may need immediate attention to complete the longstanding task of GPEI to eradicate poliomyelitis.

## Methods

**Study design:** we conducted a descriptive secondary analysis of field AFP surveillance data compiled from twenty countries of East and Southern Africa (Botswana, Comoros, Eritrea, Eswatini, Ethiopia, Lesotho, Kenya, Madagascar, Malawi, Mauritius, Mozambique, Namibia, Rwanda, Seychelles, South Africa, South Sudan, Tanzania, Uganda, Zambia, and Zimbabwe) for the period of January 2022 to December 2023. In the period, Seychelles did not report any AFP case and therefore is excluded from the analysis.

**Variables and measurement:** all variables used were obtained from the GPSAP 2022-2024 which were referred to as standard indicators. The following indicators were used to measure country performances: the NP-AFP rate, percentage of stool adequacy, percentage of districts achieved core indicators, and, timeliness in notification, investigation, field activities, and shipment of stool samples to the respective laboratories. Statistical tests were applied to evaluate the levels of statistical significance in performance improvement of the key GPSAP indicators.

**Data collection and statistical analysis:** the secondary data was collected from the AFP field database, which was shared weekly in Microsoft Access format by the data managers of countries in the ESA-Subregion. Epi Info version 3.5.4 software was used to run queries on the data, eliminate duplicate reported cases, and analyze key variables such as the country of origin, date of onset of paralysis, date of specimen collection (first and second stool samples), date of investigation, date of notification, date of receipt of stool samples at national levels and at the respective polio laboratories, and the condition of the samples received by the laboratory (good or not good).

From the primary variables mentioned secondary variables were computed and summarized in tables of results, these were the NP-AFP rate, percentage of stool adequacy, percentage of districts achieved core indicators, and timeliness in the notification, investigation, field activities, and shipment of stool samples to the respective laboratories. The Statistical Package for Social Science (SPSS) version 16 was deployed to assess the association between each variable´s performances for a similar period January to December 2022 and January to December 2023 and tested for statistical significance at a 95% confidence interval.

**Inclusion criteria for study cases:** all validated AFP cases that met the WHO standard case definition that had onset of paralysis from 1 January 2022 to 31 December 2023 and were included in the study [2]. The data source was the field AFP database for the 20 ESA countries shared on a weekly basis. A similar information may be retrieved in the polio database Polis.

**Exclusion criteria for study cases:** any other cases that are not AFP in the database or paralyzed before or after the study period, 1 January 2022 to 31 December 2023. All AFP cases and those with missing key information, for example, the date of notification, were regarded as “missing” while populating and excluded from the AFP notification tables.

## Results

A total of 18,078 AFP cases were detected, reported, and verified from nineteen countries involved in the study (Botswana, Comoro, Eswatini, Eritrea, Ethiopia, Lesotho, Kenya Madagascar, Malawi, Mauritius, Mozambique, Namibia, Rwanda, South Africa, South Sudan, Tanzania, Uganda, Zambia, and Zimbabwe) for the period 1 January 2022 to 31 December 2023. Most of the reported AFP cases, 11,141 (62%), were aged less than five years, with a median age of four years. Of the total reported AFP cases, 8,049 (44.5%) were females and 10,029 (55.5%) were males. This indicates that a male child was more likely to get AFP compared to a female child (11% difference, p < 0.0001, 95% CI ranges from 9.54% to 12.45%).

**Timeliness of notification:** we observed that out of all reported AFP cases for the two years, 11, 416 (66%) were notified within 7 days of onset of paralysis. An increase in notifications was observed from 5,452 (64%) in 2022 to 5,664 (68%) in 2023 (Table 1). This constituted an increase of 4% which was statistically significant with a p < 0.0001 (95% CI ranges from 2.59% to 5.41%). Eleven (57.9%) of countries in the ESA sub-region showed a performance improvement in early case detection (within 7 days), and these included Botswana, Eritrea, Ethiopia, Kenya, Malawi, Mozambique, South Sudan, Rwanda, Tanzania, Uganda, and Zambia. High achievements were noted in Malawi (16%), Zambia (14%), and Ethiopia (8%), while the least achievement was made in Eritrea which shows a 1% increment from 2022 to 2023. Also, we noted country variation where seven countries (Comoros, Eswatini, Lesotho, Madagascar, Namibia, South Africa, and Zimbabwe) presented a decline in performance ranging from 1% (Zimbabwe) to 20% (Comoros) for the study period. In terms of quarterly performances, an achievement of 7% increase was seen in the first quarter of 2023 when compared to the same quarter in 2022 (Table 1).

**Table 1:** distribution of the reported acute flaccid paralysis cases within 7 days of onset of paralysis by country in the ESA subregion from January 2022 to December 2023 (n=17,290)

Country Name	2022						2023					
	Q1 (%)	Q2(%)	Q3 (%)	Q4(%)	Q1-Q4 (n)	Q1-Q4 (%)	Q1 (%)	Q2(%)	Q3 (%)	Q4(%)	Q1-Q4 (n)	Q1-Q4 (%)
Botswana	0.50	0.75	0.71	0.59	34	0.62	0.62	0.75	0.60	N/A	30	0.63
Comoros	1.00	N/A	N/A	1.00	2	1.00	N/A	1.00	0.50	N/A	5	0.80
Eritrea	0.79	0.59	0.64	0.65	164	0.66	0.50	0.73	0.68	0.82	73	0.67
Ethiopia	0.64	0.66	0.58	0.61	1,366	0.63	0.67	0.71	0.72	0.73	1,187	0.71
Kenya	0.69	0.63	0.64	0.61	647	0.64	0.74	0.70	0.58	0.68	680	0.66
Lesotho	N/A	0.80	0.20	0.38	18	0.44	N/A	0.22	1.00	0.50	14	0.36
Madagascar	0.69	0.68	0.69	0.70	645	0.69	0.63	0.65	0.69	0.73	1,395	0.67
Malawi	0.52	0.41	0.41	0.63	396	0.48	0.58	0.60	0.72	0.78	408	0.64
Mauritius	N/A	0.00	1.00	0.50	6	0.50	1.00	0.50	0.00	1.00	10	0.70
Mozambique	0.38	0.44	0.44	0.62	935	0.44	0.40	0.36	0.51	0.59	668	0.46
Namibia	0.60	0.64	0.73	0.56	41	0.63	0.64	0.67	0.83	0.45	40	0.63
South Sudan	0.79	0.87	0.79	0.74	557	0.81	0.84	0.79	0.84	0.80	515	0.82
Rwanda	0.65	0.55	0.79	0.88	169	0.73	0.72	0.86	0.78	0.66	256	0.77
South Africa	0.58	0.57	0.50	0.60	445	0.56	0.55	0.51	0.53	0.50	408	0.52
Eswatini	1.00	0.50	1.00	1.00	18	0.72	1.00	0.50	0.50	0.50	9	0.67
Tanzania	0.81	0.83	0.85	0.86	1,279	0.84	0.87	0.85	0.87	0.84	1,320	0.86
Uganda	0.59	0.62	0.64	0.56	1,103	0.60	0.64	0.59	0.61	0.67	913	0.63
Zambia	0.30	0.44	0.57	0.58	376	0.46	0.49	0.65	0.62	0.79	511	0.61
Zimbabwe	0.64	0.65	0.69	0.66	327	0.66	0.63	0.61	0.64	0.74	320	0.65
**ESA average**	**0.60**	**0.64**	**0.65**	**0.67**	**8,528**	**0.64**	**0.67**	**0.67**	**0.69**	**0.70**	**8,762**	**0.68**

Note: Q1= January-March, Q2= April-June, Q3= July-September and Q4= October-December; N/A -Not Applicable

**Timeliness of investigation:** we observed that in 2023, 92% of the AFP-reported cases were investigated within 48 hours of notification. However, this indicates a 1% decline when compared to 93% in 2022. This decline is statistically significant with a p-value of 0.0129 (95% CI ranges from 0.21% to 1.79%) and a Chi-square value of 6.178. We also noted country-specific variations in timely investigation, with Eswatini, Malawi, Eritrea, Namibia, Mozambique, Madagascar, and Kenya performing well. On the other hand, delays in investigation after notification were mostly observed in Zambia, Botswana, Mauritius, and Ethiopia when compared to data from 2022 (Table 2).

**Table 2:** distribution of the acute flaccid paralysis cases investigated within 48 hours of notification by country in the ESA subregion from January 2022 to December 2023 (n=17,161)

Country name	2022						2023					
	Q1 (%)	Q2(%)	Q3 (%)	Q4(%)	Q1-Q4 (n)	Q1-Q4 (%)	Q1 (%)	Q2(%)	Q3 (%)	Q4(%)	Q1-Q4 (n)	Q1-Q4 (%)
Botswana	0.83	1.00	1.00	0.82	34	0.88	0.79	0.50	1.00	N/A	28	0.79
Comoros	1.00	N/A	N/A	1.00	2	1.00	N/A	1.00	1.00	N/A	5	1.00
Eritrea	0.97	1.00	0.91	0.86	147	0.94	0.94	1.00	0.95	0.90	69	0.96
Ethiopia	0.95	0.92	0.92	0.91	1,353	0.93	0.88	0.88	0.88	0.87	1,171	0.88
Kenya	0.99	0.91	0.94	0.98	646	0.95	0.94	0.97	0.96	0.96	680	0.96
Lesotho	N/A	1.00	1.00	1.00	18	1.00	N/A	1.00	1.00	1.00	14	1.00
Madagascar	0.93	0.98	0.94	0.95	645	0.95	0.97	0.95	0.97	0.96	1,395	0.96
Malawi	0.85	0.91	0.91	0.97	381	0.91	0.91	0.97	0.99	1.00	404	0.96
Mauritius	N/A	1.00	1.00	1.00	6	1.00	1.00	1.00	0.00	1.00	10	0.90
Mozambique	0.93	0.97	0.95	0.98	935	0.95	0.96	0.96	0.96	0.94	668	0.96
Namibia	0.90	0.91	0.82	0.89	41	0.88	0.73	1.00	0.83	1.00	40	0.90
South Sudan	0.88	0.90	0.88	0.92	557	0.89	0.86	0.92	0.87	0.85	515	0.88
Rwanda	0.97	1.00	1.00	0.98	169	0.99	0.93	1.00	0.99	1.00	256	0.98
South Africa	0.97	0.98	0.97	0.99	445	0.98	0.97	0.95	0.99	0.99	408	0.98
Eswatini	1.00	0.90	1.00	1.00	18	0.94	1.00	1.00	1.00	1.00	9	1.00
Tanzania	0.95	0.95	0.95	0.97	1,278	0.96	0.94	0.95	0.94	0.95	1,320	0.94
Uganda	0.88	0.86	0.82	0.74	1,103	0.84	0.76	0.82	0.80	0.84	903	0.81
Zambia	0.97	0.95	0.97	0.92	369	0.96	0.79	0.83	0.83	0.93	473	0.83
Zimbabwe	0.98	0.96	0.97	0.98	327	0.97	0.91	0.95	0.96	0.96	319	0.94
ESA Average	0.93	0.93	0.93	0.92	8,474	0.93	0.90	0.93	0.92	0.92	8,687	0.92

Note: Q1= January-March, Q2= April-June, Q3= July-September and Q4= October December; N/A -Not Applicable

**Timeliness of field activities:** a total of 16,036 (90%) AFP cases had two stool specimens collected within 24 hours apart and within 11 days of onset from January 1, 2022 to December 31, 2023. For this performance indicator, we noted a 2% increase from 7,790 (89%) in 2022 to 8,246 (91%) in 2023. It shows a 2% change, p < 0.0001 (95% CI ranges from 1.12% to 2.88%), and is statistically significant. Major achievements in terms of percentage increase in 2023 compared to 2022 were observed in Malawi (17%), Zambia (11%), Eswatini (10%), and Mozambique (6%). Other countries with a relative increase in achievement were Botswana, Ethiopia, South Sudan, and Rwanda. Declined performances were seen in Eritrea, Madagascar, Namibia, South Africa, Tanzania, Uganda, and Zimbabwe. The worst decline of 4% was observed in Namibia in 2023 when compared to 2022 performance, while the rest of the countries mostly had a 1% decline. No percentage changes were noted in terms of indicator performance in Comoros, Kenya, and Lesotho (Table 3).

**Table 3:** distribution of the reported acute flaccid paralysis cases with two-stool specimens collected within 24 hours apart and within 11 days of onset by country in the ESA subregion from January 2022 to December 2023 (n=17,756)

Country Name	2022						2023					
	Q1 (%)	Q2(%)	Q3 (%)	Q4(%)	Q1-Q4 (n)	Q1-Q4 (%)	Q1 (%)	Q2(%)	Q3 (%)	Q4(%)	Q1-Q4 (n)	Q1-Q4 (%)
Botswana	0.50	0.75	1.00	0.76	34	0.76	0.79	0.75	0.80	N/A	28	0.79
Comoros	N/A	N/A	N/A	1.00	1	1.00	N/A	1.00	1.00	N/A	4	1.00
Eritrea	0.92	0.93	0.91	0.90	164	0.91	0.84	0.96	0.78	1.00	76	0.88
Ethiopia	0.93	0.94	0.93	0.94	1,584	0.94	0.95	0.94	0.97	0.95	1,420	0.95
Kenya	0.90	0.88	0.88	0.81	651	0.87	0.93	0.89	0.81	0.90	693	0.87
Lesotho	N/A	1.00	1.00	1.00	18	1.00	N/A	1.00	1.00	1.00	14	1.00
Madagascar	0.96	0.93	0.96	0.97	645	0.95	0.87	0.93	0.92	0.97	1,389	0.92
Malawi	0.74	0.66	0.67	0.95	397	0.73	0.88	0.91	0.91	0.88	443	0.90
Mauritius	N/A	1.00	1.00	1.00	6	1.00	1.00	1.00	1.00	1.00	10	1.00
Mozambique	0.65	0.77	0.81	0.84	931	0.75	0.78	0.81	0.82	0.83	665	0.81
Namibia	0.60	1.00	0.91	0.88	40	0.85	0.73	0.89	0.83	0.80	36	0.81
South Sudan	0.94	0.95	0.94	0.91	557	0.94	0.96	0.93	0.95	0.98	514	0.95
Rwanda	0.85	0.98	0.97	0.98	169	0.95	0.91	1.00	0.99	0.98	256	0.98
South Africa	0.98	0.99	0.99	0.99	400	0.99	0.98	1.00	1.00	0.94	357	0.98
Eswatini	1.00	0.60	1.00	1.00	18	0.78	1.00	1.00	0.50	1.00	8	0.88
Tanzania	0.98	0.99	0.98	0.99	1,278	0.98	0.98	0.97	0.97	0.98	1,320	0.98
Uganda	0.87	0.92	0.91	0.89	1,115	0.89	0.88	0.86	0.83	0.91	943	0.87
Zambia	0.53	0.70	0.75	0.80	373	0.68	0.65	0.81	0.84	0.98	544	0.79
Zimbabwe	0.93	0.88	0.90	0.98	328	0.91	0.89	0.88	0.88	0.91	327	0.89
**ESA average**	**0.85**	**0.90**	**0.91**	**0.93**	8,709	0.89	**0.90**	**0.91**	**0.91**	**0.93**	9,047	0.91

Note: Q1= January-March, Q2=April- June, Q3=July-September and Q4=October - December; N/A -Not Applicable

**Core indicators on acute flaccid paralysis surveillance quality:** regarding the two AFP surveillance quality indicators in the study period, the average non-polio AFP rate in the ESA subregion rose from 4.4 in 2022 to 4.7 in 2023 (Table 4). This represents a statistically significant change of 0.3, p < 0.0001 (95% CI ranges from 0.234 to 0.366), and a t-statistic value of 8.880. Variation between countries exists whereby performance increment was observed from nine (47.37%) countries: Kenya, Botswana, Zambia, Tanzania, Malawi, Rwanda, Comoros, Mauritius, and Madagascar. On the other side, a decline in performance was noted in Eritrea, Ethiopia, Eswatini, Lesotho, Mozambique, Namibia, South Sudan, Uganda and Zimbabwe. Also, we noted the proportion of districts (or the first subnational levels in Botswana, Comoros, Eritrea, and Mauritius) with populations over 100,000 under fifteen years old children raised from 82% in 2022 to 83% in 2023. This indicates a one percentage change but was not statistically significant, with a p-value of 0.0847 (95% CI ranges from -0.137% to 2.138%) and a chi-square value of 2.972. Major improvements in terms of geographical improvement were observed in Zambia, Zimbabwe, Kenya, Rwanda, South Africa, Mozambique, and Madagascar while a decline was noted in Namibia, Eswatini, Lesotho, Eritrea, Ethiopia, and Uganda.

The percentage of stool adequacy during the study period increased from 89% in 2022 to 90% in 2023 in the ESA subregional average (Table 4). This indicates a statistically significant change of one percentage point, with a p-value of 0.0326 (95% CI ranges from 0.083% to 1.918%) and a Chi-square value of 4.567. We noted country variations, where Botswana, Comoros, Ethiopia, Kenya, Malawi, Mozambique, Rwanda, South Sudan, and Zambia showed improvement in the percentage of stool adequacy upon arrival into the laboratories. However, Namibia, Eswatini, Madagascar, South Africa, Eritrea, Zimbabwe, Uganda, and Tanzania indicated a decline in stool adequacy over the study period.

**Table 4:** the pattern of the non-polio acute flaccid paralysis rate, percentage of stool adequacy, and proportion of districts achieved the quality indicators’ targets by country in the ESA subregion from January 2022 to December 2023 (n=18,078)

Country	Indicator	National performance average 2022	% of districts reach the targets-2022	National performance average 2023	% of districts reach the targets-2023	National performance mean; 2022-2023	Subnational performance mean; 2022-2023
Botswana	NP-AFP rate	3.4	60%	3.6	60%	3.5	60%
	% Of stool adequacy	73%	40%	77%	80%	75%	60%
Comoros	NP-AFP rate	0.5	0%	2.5	0%	1.5	0%
	% Of stool adequacy	50%	0%	80%	50%	65%	25%
Eritrea	NP-AFP rate	9.9	80%	4.4	60%	7.1	70%
	% Of stool adequacy	91%	80%	87%	60%	89%	70%
Ethiopia	NP-AFP rate	3.4	76%	3.0	65%	3.2	71%
	% Of stool adequacy	93%	79%	95%	80%	94%	79%
Kenya	NP-AFP rate	3.2	45%	3.2	57%	3.2	51%
	% Of stool adequacy	87%	66%	88%	64%	88%	65%
Lesotho	NP-AFP rate	2.9	50%	2.4	0%	2.6	25%
	% Of stool adequacy	100%	100%	100%	100%	100%	100%
Madagascar	NP-AFP rate	5.4	95%	11.7	100%	8.5	98%
	% Of stool adequacy	95%	89%	91%	85%	93%	87%
Malawi	NP-AFP rate	5.9	100%	7.0	100%	6.4	100%
	% Of stool adequacy	72%	36%	90%	88%	81%	62%
Mauritius	NP-AFP rate	3.0	100%	5.4	100%	4.2	100%
	% Of stool adequacy	100%	100%	100%	100%	100%	100%
Mozambique	NP-AFP rate	6.3	83%	4.7	87%	5.5	85%
	% Of stool adequacy	75%	50%	80%	58%	77%	54%
Namibia	NP-AFP rate	4.0	100%	2.6	0%	3.3	50%
	% Of stool adequacy	83%	100%	71%	0%	77%	50%
Rwanda	NP-AFP rate	2.9	83%	4.8	90%	3.9	87%
	% Of stool adequacy	95%	100%	97%	100%	96%	100%
South Sudan	NP-AFP rate	8.8	100%	8.2	100%	8.5	100%
	% Of stool adequacy	94%	93%	95%	96%	94%	94%
South Africa	NP-AFP rate	2.5	58%	2.6	67%	2.6	62%
	% Of stool adequacy	89%	73%	85%	71%	87%	72%
Eswatini	NP-AFP rate	4.8	100%	2.4	50%	3.6	75%
	% Of stool adequacy	75%	50%	70%	50%	73%	50%
Tanzania	NP-AFP rate	4.9	95%	6.0	96%	5.5	95%
	% Of stool adequacy	98%	97%	98%	95%	98%	96%
Uganda	NP-AFP rate	5.0	90%	4.3	88%	4.7	89%
	% Of stool adequacy	90%	88%	87%	76%	88%	82%
Zambia	NP-AFP rate	4.6	61%	5.5	83%	5.1	72%
	% Of stool adequacy	66%	50%	78%	72%	72%	61%
Zimbabwe	NP-AFP rate	5.1	68%	4.7	79%	4.9	73%
	% Of stool adequacy	91%	96%	88%	82%	90%	89%
East and Southern Africa average	NP-AFP rate	4.4	82%	4.7	83%	4.6	83%
	% Of stool adequacy	89%	81%	90%	81%	89%	81%

Regarding subnational performance evaluation of stool adequacy, we noted no variation for the two years of 2022 and 2023 at the ESA sub-regional level. However, variations exist between countries, with increased performance observed in Malawi, Comoros, Botswana, Zambia, Mozambique, South Sudan, and Ethiopia, while a decline was observed in Namibia, Eritrea, Zimbabwe, Uganda, Madagascar, Tanzania, Kenya, and South Africa (Table 4).

**Timeliness of stool specimen shipment:** a total of 17,630 AFP cases stool samples were assessed and we noted that 53% were delivered to the national polio laboratories within 72 hours during the study period. We noted an increase in the proportion of stool samples that were delivered within 72 hours to the polio laboratories from 52% in 2022 to 53% in 2023 (Table 5). This shows a one percentage change which is not statistically significant, with a p-value of 0.1838 (95% CI ranges from -0.474% to 2.474%) and a Chi-square value of 1.767.

**Table 5:** analytical summary of stool specimens from acute flaccid paralysis cases collected within the three days following the collection, to WHO-accredited laboratories by country in the ESA sub-region, from January 2022 to December 2023 (n=17,630)

Country Name	2022						2023					
	Q1 (%)	Q2(%)	Q3 (%)	Q4(%)	Q1-Q4 (n)	Q1-Q4 (%)	Q1 (%)	Q2(%)	Q3 (%)	Q4(%)	Q1-Q4 (n)	Q1-Q4 (%)
Botswana	0.33	0.00	0.14	0.35	34	0.26	0.20	0.00	0.17	N/A	30	0.17
Comoros	N/A	N/A	N/A	0.00	1	0.00	N/A	0.00	0.00	0.00	9	0.00
Eritrea	0.00	0.00	0.00	0.00	147	0.00	0.00	0.00	0.00	0.00	56	0.00
Ethiopia	0.98	0.98	0.98	0.99	1,584	0.98	0.96	0.97	0.89	0.96	1,443	0.95
Kenya	0.84	0.84	0.89	0.90	651	0.86	0.90	0.88	0.94	0.91	695	0.91
Lesotho	N/A	1.00	1.00	0.75	18	0.89	N/A	0.67	0.00	0.00	14	0.43
Madagascar	0.68	0.73	0.73	0.73	645	0.72	0.60	0.51	0.41	0.50	1,395	0.49
Malawi	0.02	0.02	0.00	0.00	375	0.01	0.02	0.22	0.31	0.13	393	0.18
Mauritius	N/A	0.00	0.00	0.25	6	0.17	0.00	0.00	1.00	0.00	11	0.09
Mozambique	0.02	0.03	0.14	0.13	930	0.06	0.25	0.22	0.23	0.36	665	0.25
Namibia	0.22	0.18	0.18	0.14	38	0.18	0.10	0.13	0.33	0.00	31	0.13
South Sudan	0.00	0.00	0.00	0.00	557	0.00	0.00	0.01	0.00	0.06	458	0.00
Rwanda	0.18	0.10	0.08	0.09	169	0.11	0.35	0.48	0.37	0.46	256	0.42
Seychelles	N/A	N/A	N/A	N/A	N/A	N/A	N/A	N/A	N/A	N/A	N/A	N/A
South Africa	0.57	0.62	0.63	0.58	400	0.60	0.64	0.55	0.60	0.60	358	0.62
Eswatini	1.00	0.40	0.67	0.75	18	0.56	0.50	0.67	0.00	0.00	9	0.56
Tanzania	0.00	0.00	0.00	0.02	1,278	0.01	0.01	0.01	0.08	0.08	1,330	0.04
Uganda	0.90	0.87	0.90	0.83	1,115	0.88	0.84	0.79	0.78	0.78	952	0.78
Zambia	0.95	0.90	0.85	0.76	373	0.87	0.81	0.80	0.78	0.78	543	0.81
Zimbabwe	0.91	0.73	0.74	0.84	327	0.78	0.79	0.69	0.85	0.85	316	0.78
**ESA** average	0.55	0.51	0.48	0.55	8,666	0.52	0.51	0.48	0.51	0.51	8,964	0.53

Note: Q1= January-March, Q2= April-June, Q3= July-September and Q4= October-December; N/A -Not Applicable: ESA: East and Southern Africa

It was observed that there were differences in the way shipments were being handled across various countries. Improvements were made in the shipment process in Rwanda, Mozambique, Malawi, Kenya, Tanzania, and South Africa, while the shipment process declined in Ethiopia, Namibia, Zambia, Mauritius, Botswana, Uganda, Madagascar, and Lesotho. Additionally, it was noted that there were quarterly variations, where most stool samples arrived beyond 72 hours in the respective polio laboratories during the second quarter in 2023 and the third quarter in 2022 (Table 5).

## Discussion

In our study, we noted that in some countries, delayed notification contributed to the delays of the entire field surveillance timeliness and quality indicators. The previous studies in the ESA sub-region indicated improvement in the core quality surveillance indicators over the period before the COVID-19 pandemic and declined performance in 2020 in the COVID-19 pandemic peak seasons [15,16]. We noted that the countries that were performing well before the COVID-19 pandemic, during the pandemic, and after post-pandemic continue to be the same. Also, countries with outbreaks such as Malawi, Botswana, Zambia, Mozambique, and Rwanda had shown the impact of the surge support in the period while Madagascar, Tanzania, and Zimbabwe had not shown a relativity of performance improvement in GPSAP indicators. We also noted a dramatic performance decline in Namibia, Eritrea, Uganda, and South Africa over the study period, and therefore a special intervention would be advisable to accelerate the reversal of the adverse trend among these countries.

We have observed an improvement in the timely notification of cases, which is a positive development towards achieving the key indicators of the GPSAP 2022-2024. This improvement helps in detecting and investigating cases promptly, with stool samples collected for confirmation and response, as necessary. Countries with weaker surveillance structures and systems require interventions such as community sensitization and engagement, including community-based healthcare service delivery points. In countries facing civil unrest or instability, the use of community informants may be an innovative way to increase and sustain the AFP case detection rates.

Also, we noted that the improvement of field surveillance timeliness in the subregion was an achievement attributable to the various trainings, weekly performance feedback, and support given by the polio surge teams in some countries with outbreaks. We suggest a critical review to document the impact of availing and removing polio surge support before and after outbreak response in countries to contribute towards the sustainable strategic approaches of the achievements made at the heightened support. The observation for field surveillance timeliness in good and sub-optimally performing countries showed similar findings. Nonetheless, we also noted the need for cascading initiatives applied to countries at national levels to the subnational levels since a sizable number of countries that showed good performances at national averages also had performance gaps at subnational levels.

In the last quarter of 2023, almost all indicators showed improvement, which could be attributed to the physical, face-to-face capacity building conducted at the sub-regional and regional levels. Due to the COVID-19 pandemic, virtual training was mostly used for capacity building, including GPSAP orientation. Previous studies have suggested that virtual realities are as effective as physical realities in training and simulations, especially in low-resourced countries that may be used in the last miles for the polio eradication [17]. These findings seem to be more conclusive if applied in a controlled setting, such as with medical students, where long-term memory retention has been observed to be reliable. However, for adults, individual motivation and perceived value of the training remain questionable due to divided attention while attending virtual meetings [18,19]. In this regard, a mix of face-to-face and virtual reality may be an option for capacity building for vaccine-preventable diseases (VPDs) to see sustained impact towards and after the eradication goal achievement.

**Study limitations:** we were not able to predict some indicators in countries where the dates were missing in the shared database. In addition, any information on AFP cases delayed being reported by 31 January 2024 was not included in the study. We did not cover the laboratory and ES indicators, a substantial proportion of results for 2023 were still in the laboratory, and by 31 January 2024, the available data could not be sufficient to reflect the reality of the year. Also, in Botswana, Comoros, Eritrea, and Mauritius because of the low population at the lowest administrative levels, we used the first subnational level for calculating quality indicators for geographical distribution. Countries have recovered from the effects of the COVID-19 pandemic with different support mechanisms - and these are not well known.

## Conclusion

There is an overall improvement in the GPSAP performance indicators in the ESA subregion for the period of 1 January 2022 to 31 December 2023. There is also performance improvement in the early detection of AFP cases within 7 days of onset of paralysis, timely investigation after notification, timeliness of AFP surveillance activities within 11 days of onset of paralysis in the ESA sub-region, and shipment of stool specimens within 72 hours. We noted countries' variations and suggested the use of innovations in AFP case detections, cascading surveillance training to lower levels, and improved shipment of samples by increasing the number of polio laboratories with capacities to process stool samples. We also noted gaps in some countries especially at subnational levels and advice on the critical surveillance structures reviews to determine and mitigate identified issues. More efforts are needed to ensure all key field performance indicators are achieved in the highlighted countries and maintained towards polio eradication and beyond.
